# The Action of Chloroform on the Blood

**Published:** 1915-01-09

**Authors:** 


### The Action of Chloroform on the Blood.

Dr. J. W. B. Bean, a Cambridge graduate living in the Antipodes, has given to the *Australasian Medical Gazette* the result of his experiments to determine whether Mr. Richard Gill's hypotheses concerning the manner in which chloroform brings about anæsthesia are correct. Mr. Gill, the senior anæsthetist at St. Bartholomew's Hospital, holds that chloroform acts by depriving the nervous centres of oxygen—*i.e.*, by causing asphyxia of the nerve cells. Dr. Bean says that, on the contrary, this is not the case. Chloroform is poisonous to the blood; in low percentages slowly killing it, in higher strengths quickly laking it. He thinks it quite possible that when chloroform is given in minimal doses (just enough to produce anæsthesia) it remains confined to the blood, not invading the tissues generally. It may thus cause an oxygen starvation of the tissues and brain indirectly by combining with the red cells and temporarily lessening their power of taking up oxygen and handing it on to the tissues. This, however, is different to ordinary asphyxia in that there is no blockage of the pulmonary circulation, whereas in typical asphyxia there is such a blockage. Hence anæsthesia does not cause, according to this view, a tissue asphyxia; unless, of course, cyanosis and asphyxia be allowed to supervene through faulty administration.

